# Nitric oxide metabolites in the lumbosacral spinal cord interstice and cerebrospinal fluid in female rats with acute cyclophosphamide-induced cystitis. An *in vivo* microdialysis study

**DOI:** 10.1590/S1679-45082013000100016

**Published:** 2013

**Authors:** Jeova Nina Rocha, Gustavo Ballejo

**Affiliations:** 1 Universidade de São Paulo, Ribeirão Preto, SP, Brazil

**Keywords:** Cystitis/chemically induced, Nitric oxide, Microdialysis, Cyclophosphamide, Omega-N-methylarginine

## Abstract

**Objective:**

To determine the concentration of nitrate/nitrite in the cerebrospinal fluid and in the dorsal horn interstice of the L6-S1 spinal cord boundary in rats with or without cystitis induced by cyclophosphamide.

**Methods:**

All experiments were conducted using Wistar female rats. A microdialysis probe was implanted in the subarachnoid space or in the spinal cord tissue at the L6-S1 segments (confirmed histologically). Two days later, the microdialysis probe was perfused with artificial cerebrospinal fluid, containing or not NGmonomethyl-L-arginine. Samples were collected every 15 minutes and kept at −20°C. Nitrite/nitrate concentrations were determined by chemiluminescence.

**Results:**

In normal animals, the mean values of nitrite/nitrate concentrations in the first microdialysate sample of the cerebrospinal fluid and of the spinal cord interstice were similar (482.5±90.2pmol/75μL, n=20, and 505.7±11.5pmol/75μL, n=6, respectively), whereas, in the samples from rats with cystitis, these values were significantly greater (955.5±66.3pmol/75μL, n=8, and 926.5±131.7pmol/75μL, n=11, respectively). In both groups, NGmonomethyl-L- arginine caused a significant reduction in the nitrite/nitrate concentration. Interestingly, the maximal reduction of nitrite/nitrate concentration caused by NG-monomethyl-L- arginine was no greater than 30% of the initial values.

**Conclusions:**

These results constitute the first demonstration that nitrite/nitrate concentrations in the cerebrospinal fluid and spinal cord interstice are elevated between 20- and 22 hours after cyclophosphamide-induced cystitis, and indicate that cystitis is associated with changes in the production of nitric oxide in the spinal cord segments, where most primary bladder afferents end.

## RESUMO

**Objetivo:**

Determinar a concentração de nitrato/nitrito no líquido cefalorraquidiano e no interstício do corno dorsal entre L6-S1 da medula espinhal em ratas com ou sem cistite induzida por ciclofosfamida.

**Métodos:**

Todos os experimentos foram conduzidos usando ratas Wistar. Um probe de microdiálise foi implantado no espaço subaracnoide ou no tecido da medula espinhal nos segmentos L6-S1 (confirmado histologicamente). Dois dias depois, o probe de microdiálise foi perfundido com líquido cefalorraquidiano artificial, contendo ou não NG-monometil-L-arginina. As amostras foram coletadas a cada 15 minutos e mantidas a −20°C. As concentrações de nitrito/ nitrato foram determinadas por quimiluminescência.

**Resultados:**

Nos animais normais, os valores médios das concentrações de nitrito/nitrato, na primeira amostra de microdialisado de líquido cefalorraquidiano e do interstício da medula espinhal, foram semelhantes (482,5±90,2pmol/75μL, n=20, e 505,7±11,5pmol/75μL, n=6, respectivamente), enquanto nas amostras de ratas com cistite, esses valores foram significativamente maiores (955,5±66,3pmol/75μL, n=8, e 926,5±131,7pmol/75μL, n=11, respectivamente). Em ambos os grupos, a NG-monometil-Larginina causou uma significativa redução na concentração de nitrito/nitrato. Curiosamente, a redução máxima de concentração de nitrito/nitrato causada pela NG-monometil-L- arginina não foi maior que 30% dos valores iniciais.

**Conclusões:**

Esses resultados constituem a primeira demonstração de que as concentrações de nitrito/nitrato no líquido cefalorraquidiano e no interstício da medula espinhal estão elevadas entre 20 e 22 horas após a cistite induzida por ciclofosfamida, e indicando que a cistite está associada a alterações na produção de óxido nítrico, nos segmentos da medula espinhal, nos quais termina a maioria dos aferentes primários da bexiga.

## INTRODUCTION

The function of the lower urinary tract is to store and eliminate urine. This function is regulated by a neuronal complex mechanism involving cholinergic neuroeffector neurotransmitters (detrusor, urethra striated muscle, and ganglionic synapses), adrenergic and nitrergic neurotransmitters (urethral smooth muscle), as well as glutaminergic and peptidergic neurotransmitters at the spinal and supraspinal levels^(1)^. Evidence has been accumulated, especially from pharmacological studies using nitric oxide synthase (NOS) inhibitors, indicating that NO- or NOS-dependent neurotransmission in lumbosacral spinal segments is implicated in the hyperreflexia of the detrusor associated with bladder inflammation^(2–5)^. However, a direct documentation of the production/release of NO in the spinal cord segments receiving primary afferents from the urinary bladder in animals with chemically-induced cystitis is lacking.

Consequently, the experiments of the present study were conceived to answer the following questions:

– are NO metabolites present in the cerebrospinal fluid or in the spinal cord interstice at the L6-S1 boundary level in normal conditions?
– is cyclophosphamide (CYP)-induced cystitis associated with any significant change in the amount of these metabolites present in the spinal cord interstice or cerebrospinal fluid?
– are NO-metabolite levels in spinal cord interstice or cerebrospinal fluid modified by L-N-monomethyl arginine (L-NMMA), an inhibitor of NOS synthase, in both conditions?

## OBJECTIVE

To determine the concentration of nitrate/nitrite in the cerebrospinal fluid and in the dorsal horn interstice of the spinal cord L6-S1 boundary in rats with or without cystitis induced by cyclophosphamide CYP.

## METHODS

All experiments were performed using Wistar female rats weighing 230- to 260g, maintained in special boxes with a synchronized light/dark cycle of 12/12 hours, at temperatures of 24±2.0°C. The experiments were approved and performed in accordance with guidelines for animal experiments approved by the Committee of Ethics in Animal Experimentation of the Faculdade de Medicina de Ribeirão Preto of Universidade de São Paulo (USP), protocol number 028/2004). The animals received an injection of CYP 150mg/kg, intraperitoneal (ip), under ethyl-ether anesthesia, 20 to 22 hours before the microdialysis procedures, and were placed in special boxes, with no drinking water for the first 12 hours. All experiments were conducted in the Laboratory of Neurology of Faculdade de Medicina de Ribeirão Preto da Universidade de São Paulo (USP).

The probe for microdialysis was prepared in our laboratory, using a tubular fiber (Filtral AN 69, microdialysis 200μM, Hospital Industrie, Gambro), measuring 6.0cm in length coated with a thin layer of epoxy, except for a 2-mm uncoated portion in the middle that was considered the effective zone for dialysis. The procedures for implanting the microdialysis probe were modified from those described by Sluka and Westlund^(6)^ and were described in abstract form previously^(7)^. Briefly, the animals were anesthetized with 2.5% tribromoethanol (0.25mg/g body weight, ip, supplemented with doses of 0.1mg/g body weight, if necessary). Using a dental drill (with a 300μM diameter steel burr), a small hole was made in the laminae of the L2 vertebra, taking care to avoid damage to the spinal cord. The probe was introduced through these holes in order to be implanted in the interstice of spinal cord L6-S1 segment boundary (to dialyze the dorsal horn and the dorsal commissure) or in the subarachnoid space (L6-S1 level) and fixed to the vertebral laminae with cyanoacrylate. Both ends of the probe were attached to a polyethylene tubing (PE-20, internal diameter=0.38mm; external diameter=1.09mm, Clay Adams, Parsippany, NJ, USA) and glued with cyanoacrylate. These tubes were exteriorized through a subcutaneous tunnel in the dorsal cervical area of the animal. PE tubes and probe were filled with artificial cerebrospinal fluid (aCSF) containing 10% heparin, and the external tips were sealed with cautery. After surgery, a deep intramuscular injection of penicillin (8,000IU/animal) was administered. The microdialysis was performed 2 days after implantation of the probe in the animals anesthetized with urethane,1.0g/kg, administered sub cutaneously (sc). Body temperature of the animals was continuously monitored with a thermal microsensor placed in the rectum, and was maintained at 37°C during the experiment with a heating blanket (CMA/150 – Temperature Controller).

**Figure 1:**
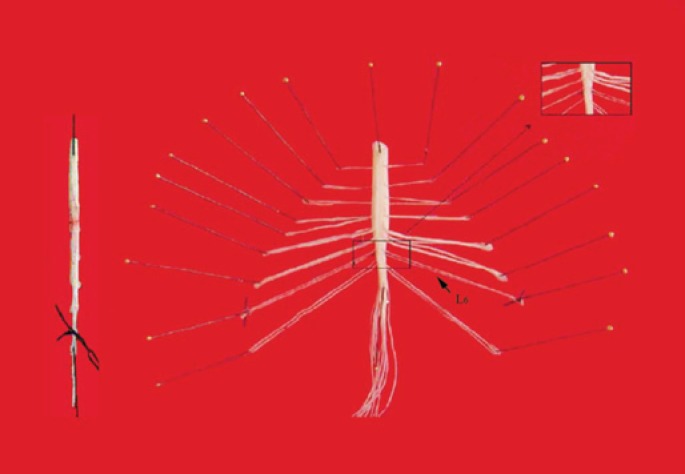
Demonstration of the technique used to isolate and identify the spinal cord segment L6-S1, in which where the probe was implanted. The left figure corresponds to the spinal cord soon after its removal. The black line of this figure identifies afferent/efferent fibers of L6. In the central figure, lateral arrows identify the spinal nerves L5, L6, S1, and the rectangle in the spinal cord indicates the site of implantation of the microdialysis tube (microdialysis zone) in this segment. The figure at the right corresponds to a magnification of the probe implantation site

Fluorinated ethylene propylene (FEP) cannulae previously perfused with 30% ethanol (30 to 40 minutes) and washed with Mill-Q water for 20 minutes were connected to the exteriorized PE tubes and aCSF (composition in g/L: NaCl=8.1; KCl=0.25; Na2HPO4=0.08; MgCl26H20=0.1; NaHC03=1.72; CaCl2 2H20=0.14; (NH2)2CO=1.3; Glucoseglucose=0.6, pH=7.4, previously gassed with 95% O_2_/ 5% CO_2_) which was perfused (5μL/minute) for 30 to 40 min minutes to establish a diffusion equilibrium. Following this stabilization period, samples of 75μL of the dialysate were collected (every 15 minutes) in special plastic tubes with an automatic collector (microdialysis collector CMA/142) and kept at −20°C until their analysis. At the end of the experiment, the animals were perfused with 500mL of ice-cold saline containing heparin (1.0IU/mL) and sodium nitrite (1.0mg/mL), through a needle placed in the ascendant aorta, for 10 to 15 minutes, at a flow rate of 40 to 45mL/minutes; the animals were then perfused with a solution of 4% buffered paraformaldehyde (in phosphate buffered saline, PBS) for another 45 minutes (flow rate 25mL/minutes). The bladder was removed and processed for conventional histological analysis and histochemistry for NADPH diaphorase as described in our a previous publication^(8)^. The spinal cord was sectioned at the mid-thoracic region and the lower part (including the lower thoracic and lumbosacral segments as well as the cauda equina) was removed *en bloc* and placed on a Sylgard-coated plaque; the fibers of the cauda equina were identified and isolated in order to locate the L6-S1 segments (Figure 1). This segment was then dissected out and post-fixed in the same fixative for 2 to 4 hours. For immunohistochemistry (IHC) determinations, this segment was embedded in paraffin and transverse sections of 6.0μm were processed for neuronal NOS (nNOS) IHC(see below). A set of normal rats without an implanted probe were also perfused in the same manner, had their spinal cord removed and post-fixed similarly, but cryo-preserved in 30% sucrose at 4°C for 24 hours and embedded in tissue-tek and stored at −70°C until processed for reduced nicotinamide adenine dinucleotide phosphate–diaphorase (NADPHdiaphorase) histochemistry.

### Histochemical determination of NADPH diaphorase and IHC determination of neuronal NOS (nNOS)

The procedures for histochemical and IHQ determinations were modified from those described in a previous publication^(5)^. Briefly, cryostatic (-17°C) transverse sections (40μm) were incubated (free-floating) in 0.1M phosphate buffer (pH pH=7.4) containing 0.35% triton X-100, 0.1mg/mL nitroblue tetrazolium (Sigma), and 1.0mg/mL β-NADPH (Sigma) at 37°C for 1 hour. Sections were rinsed with 0.1M phosphate buffer, mounted on gelatinized slides and covered with Permount (Fisher Scientific, Fair Lawn, NJ, USA).

### nNOS IHC

Paraffin-embedded sections (6.0μm) mounted on slides were pre-incubated with 1% H2O2 in potassium phosphate buffered saline (KPBS) for 10 minutes. After washing, the sections were incubated with a blocking solution (10% non-immune goat serum) for 30 minutes. Sections were then incubated with the primary antibody (rabbit polyclonal anti-nNOS SC R-20) 0.1μg/mL in 0.1M KPBS containing 5% goat non-immune serum and 0.3% triton X-100 for 16-20 hours at room temperature under gentle shaking. After extensive washing with KPBS, sections were incubated with biotinylated goat anti-rabbit IgG (1.0mg/mL) for one 1 hour and after washing, further incubated with 1mg/mL of ABC complex (avidin-biotin-horseradish peroxidase, Vector Laboratories Inc., Burlingame, CA, USA) for 1 hour. Staining was visualized with 3,3′diaminobenzidine tetrahydrochloride (Vector Laboratories, Inc). Sections were rinsed and cover-slipped with Permount. Negative controls consisted of assays in which the primary antibody was omitted in the incubations. Sections were observed under an Olympus BX50 microscope and high-resolution images (4140x3096 pixels) were captured with an Olympus DP72 camera and digitized using specific software (Image Pro-Plus 6.0 from Media Cybernetics, Silver Springs, MD, USA).

### Determination of nitrite/nitrate concentrations (NOx)

**Figure 2:**
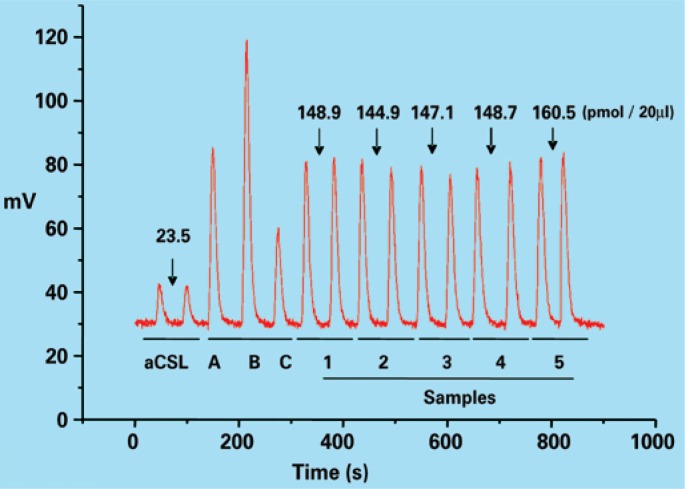
Representative original tracing of the chemiluminescence signals corresponding to NOx metabolites present in 20μL of microdialysis samples obtained from a urethane-anesthetized female rat with a microdialysis probe implanted in the spinal cord segment (L6-S1) and continuously perfused with artificial cerebrospinal fluid (aCSF) (5μL/min). A, B, and C correspond to the signals produced by nitrate standard concentrations. Values shown on top of the signal peaks represent the average concentration (pmol/20μL) of NOx metabolites (nitrite/nitrate) present in the samples calculated from the area of each peak. Each sample was assayed in duplicate. The signals produced by 20μL of the aCSF are also shown

The quantification of NOx metabolites present in the samples was performed by chemiluminescence using a NO analizer (NOA, Sievers). Briefly, samples were added to a reaction chamber containing a saturated solution of vanadium (III) chloride (VCl3) in HCl (1.0 M), maintained at 95°C and continuously purged with nitrogen. Nitrogen carries the NO formed in the reaction of nitrite/nitrate with VCl3 first to a NaOH solution, which retains the chlorine gas, then to the ozone chamber at −18°C, where in which the chemiluminescence reaction occurs. This procedure detects NO derived from both nitrite and nitrate as well as from nitrosothiols and other compounds containing a nitro group (nitro-arginine, for instance). After establishing the values of known concentrations (using 20μL of nitrate solutions of different concentrations 1, 3, 10, 20, 30μM), the NOx concentrations present in 20μL of the aCSF and of the microdialysate samples were determined in duplicate. The concentration of NOx present in the samples was calculated using the linear regression equation relating the standard concentrations and the area of the peaks (Figure 2). The concentration of NOx present in the aCSF was subtracted from the values of the samples and the final NOx values present in the microdialysate samples were expressed inpmol/75μL (equivalent topmol/15 minutes). Values of NOx present in the aCSF were 40.0±2.5pmol/75μL (mean±SEM).

Experiments with the probe in the subarachnoid space were conducted in normal animals (without cystitis) with no L-NMMA added (n=7) or with 100μM L-NMMA added (100μM) (n=6) or (300μM) (n=7) to the dialyzing solution as well as in animals with CYP-induced cystitis (n=8). Experiments with the probe in the interstice of segments L6-S1 were conducted in normal animals (n=6) and in animals with CYP-induced cystitis with no LNMMA added (n=5) or with the addition of 100μM L-NMMA (100μM) (n=6) to the dialyzing solution. Only NOx values from microdialysate samples from animals that had the probe transversally implanted between 200 and 250μM dorsally to the central canal of the spinal cord were considered for analysis.

The concentration of NOx of the first sample of the microdialysate was expressed inpmol/75μL. For the time-course analysis, NOx concentrations, either in the CSF or in the spinal cord interstice, were expressed as percentages of the value observed in the first sample. The statistical evaluation of the changes observed during the time course of the experiments or after the addition of L-NMMA to the dialyzing solution was performed using one-way ANOVA followed by Dunnett's multiple comparison test; and the statistical significance was defined as p<0.05. The mean±SEM of NOx concentration present in the first sample of CSF or interstice microdialysates was compared between normal and CYP-induced cystitis animals; the statistical significance of the differences was determined using the two-tailed Student *t* test for independent samples; p<0.05 was considered significant. The drugs used in this study included tribromoethanol and sodium nitrate (Aldrich Chemical Co.), urethane, and cyclophosphamide CYP (Sigma-Aldrich Co.), NG-Monomethylmonomethyl-L-Arginine arginine (L-NMMA – Peninsula Lab Inc.), procaine penicillin (Lab Teuto), and heparine (Cristalia S.A.).

## RESULTS

On the day of the microdialysis (20 to 22 hours after CYP administration), CYP-treated animals showed gross hematuria; the histopathological analysis of the bladder from these animals, removed after performing the microdialysis, showed an intense hemorrhagic inflammation on the bladder wall (data not shown).

**Figure 3:**
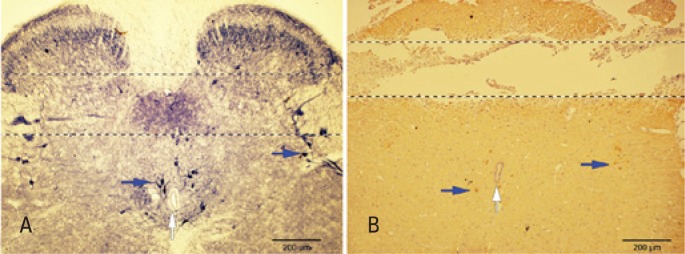
(A). Microphotography of a transversal cryostatic section (40μM) of the spinal cord segment (L6-S1) showing NADPH-diaphorase-positive neurons (blue arrows) and neuropil. Discontinued lines show the site where where the probe was located in the experiments in which the interstice tissue was microdialyzed. It can be noted that the probe position is approximately 200μM dorsally to the central canal (white arrow). (B). Microphotography of a transverse section (6μM) obtained from a paraffin-embedded spinal cord segment (L6-S1) showing positive nNOS immunoreactive neurons (blue arrows) and fibers as well as the tunnel left by the microdialysis probe. Note that the tunnel is approximately 200μM dorsally to the central canal (blue arrow)

As can be observed in figure 3B, the position of the microdialysis probe in the spinal cord is revealed by the tissue discontinuity easily apparent in the section processed for nNOS IHQ; in this representative section, the probe was in a perfect transversal orientation about 200μM dorsally to the central canal; as shown in figure 3A, this region includes the parasympathetic sacral and dorsal commissural nuclei which contain numerous NADPH-diaphorase-positive neurons and fibers. It is also possible to observe the occurrence dorsally to the probe of NADPH-positive fibers and a few small neurons located in the most superficial laminae of the dorsal horn. In figure 3B, neurons and fibers exhibiting positive immunoreactivity for nNOS are seen in the parasympathetic sacral nucleus and around the central canal.

**Figure 4:**
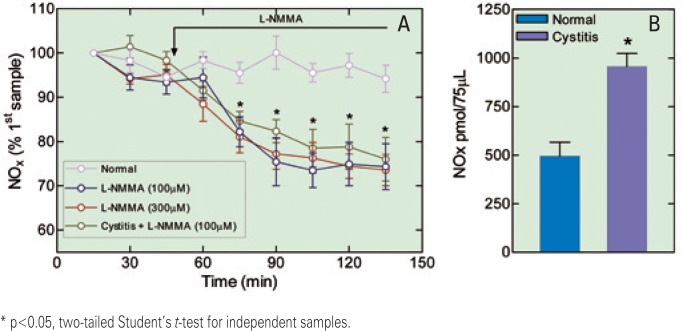
(A) Time course of nitric oxide metabolites (NOx) concentrations (mean±SEM) present in samples obtained through a microdialysis probe implanted in the subarachnoid space (segment level L6-S1) from a group of normal rats (normal), a group of normal rats in which NG-monomethyl-L-arginine (L-NMMA, 100μM) was added to the dialyzing artificial cerebrospinal fluid (aCSF), a group of normal rats in which L-NMMA (300μM) was added to the dialyzing aCSF, and a group of rats with cyclophosphamide-induced cystitis in which L-NMMA (100μM) was added to the dialyzing aCSF. (B). Bar graph showing the mean±SEM (normal n=20 and cystitis n=8) of NOx concentrations present in the first sample of the CSF microdialysate

The concentration of NOx in the first sample of the CSF microdialysate from all normal animals was 482.5±90.22pmol/75μL (mean±SEM; n=20). During the elapsed experimental time (135 minutes), these values showed no significant change (p>0.5, one-way ANOVA) (Figure 4). In normal animals, there was a significant reduction in the NOx concentration 30 minutes after L-NMMA (100 or 300μM) was added to the aCSF (p<0.05, one-way ANOVA, Dunnett's multiple comparison test). L-NMMA (300μM) also caused a reduction in NOx concentration 30 minutes after its addition (p<0.05, one-way ANOVA, Dunnett's multiple comparison test), but interestingly, the maximal reduction was not different from the one caused by 100μM (Figure 4). The concentration of NOx in the first sample of the microdialysate from animals with CYP-induced cystitis was 955.5±66.3pmol/75μL (mean±SEM; n=8); this value was significantly greater than the corresponding value in normal animals (p<0.05). In these animals, the addition of L-NMMA (100μM) to the dialyzing aCSF also caused a significant reduction in the concentration of NOx (p<0.05; one-way ANOVA, Dunnett's multiple comparison test) (Figure 4). Intriguingly, even after the inhibitor had been perfused for 90 minutes, the maximal reduction of the NOx concentration was no greater than 30% of the initial values in both normal animals and those with CYP-induced cystitis.

**Figure 5:**
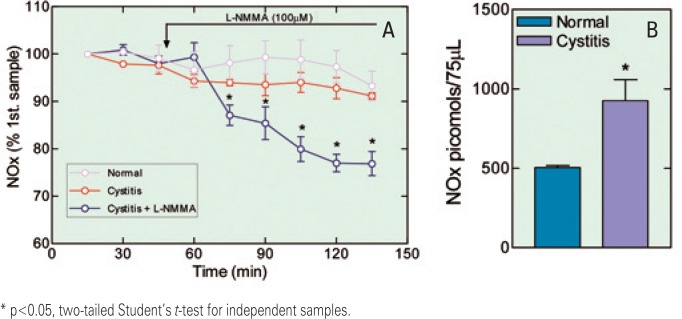
(A) Time course of nitric oxide metabolites (NOx) concentrations (mean±SEM) present in samples obtained through a microdialysis probe implanted in the spinal cord dorsal horn (segment level L6-S1) from a group of normal rats (normal), a group of rats with cyclophosphamide (CYP)-induced cystitis (cystitis), and a group of rats with CYP-induced cystitis in which NGmonomethyl-L-arginine (L-NMMA, 100μM) was added to the dialyzing artificial cerebrospinal fluid (aCSF) (cystitis + L-NMMA). (B). Bar graph showing the mean±SEM (normal n=6 and cystitis n=11) of NOx concentrations present in the first sample of the dorsal horn microdialysate

The concentration of NOx in the first sample of the microdialysate from the spinal cord interstice was 505.7±11.5pmol/75μL (mean±SEM; n=6) in normal animals and 926.5±131.7pmol/75μL (mean±SEM; n=11) in animals treated with CYP; this difference was statistically significant (p<0.05). During the time elapsed (135 minutes), these values did not change significantly (Figure 5). The concentration of NOx in animals with CYP-induced cystitis decreased significantly 30 minutes after adding L-NMMA (100μm) to the dialyzing aCSF; it is interesting to note that this reduction was no greater than 30% of the value of the initial samples, even after 90 minutes of perfusion with aCSF containing the NOS inhibitor, as is observed with the NOx values of the subarachnoid fluid, no greater than 30% of the value of the initial samples, even after 90min of perfusion with aCSF containing the NOS inhibitor (Figure 5).

## DISCUSSION

The present study constitutes the first direct demonstration that NOx is increased both in the CSF and in the dorsal horn interstice of spinal cord segments L6-S1 boundary 20 to 22 hours after CYP administration, which causes a hemorrhagic cystitis. Previous studies in which similar determinations were performed include: (1) – one study in which the NOx concentration in microdialysate samples of the spinal cord interstice was determined after the injection of capsaicin in one hind paw^(9)^; (2) – one study in which the NOx concentration in the microdialysate of CSF was determined after an intrathecal injection of NMDA^(10)^; (3) – one study in which the NOx concentration in the interstice of the dorsal horns (L4 segment) was determined in animals after subcutaneous formalin or zymosan injections in one hind paw^(11)^; and (4) – one study in which the NOx concentration in CSF microdialysate was determined after high-dose intrathecal morphine administration^(12)^. As the hemorrhagic cystitis caused by CYP is associated to intense bladder hyperreflexia^(13)^, the results of the present study suggest that the observed increased production of NO in L6-S1 segments might contribute to this hyperreflexia. Indeed, bladder hyperreflexia associated with inflammation or irritation of the bladder involves a central sensitization (at the segmental level) that has been proposed to be dependent on NO synthesis^(2–5)^ on the basis of the effects of drugs that inhibit NOS synthase activity. Consequently, the findings of the present study, in which determinations of NO production (through measurements of its stable metabolites) in the interstice of L6-S1 segments boundary were obtained and where most bladder primary afferents end^(14)^, constitute direct evidence for the postulated increase in NO-mediated spinal neurotransmission in animals with chemically induced cystitis. It is not unlikely that the NO metabolites measured in the interstice reflect predominantly NO produced by neurons, since the probe was positioned in the area adjacent to the sacral parasympathetic nucleus and dorsal commissural nucleus, where in which there is a great density of neurons containing NOS; we cannot discard, however, some contribution from glial and endothelial cells present in the spinal cord, which are also able to produce NO. It is also possible that a fraction of the NO could have originated from some NOS-positive neurons present in the dorsal horn. Regarding NO metabolites present in the CSF, and taking into account that their concentrations were similar to those found in the spinal cord interstice, it is most likely that they originate from the NO metabolites present in the interstice that had diffused to the CSF; furthermore these findings reveal the existence of a diffusional steady state of NOx concentrations between both compartments. We also cannot discard the possibility that some NOx could have originated from NOS-positive neurons found in the most superficial layers of the L6-S1 segment dorsal horns, or from neurons located more cranially in the CNS.

Considering that the three isoforms of NOS capable of forming NO are present in spinal cord cells^(15)^, another question arises: which NOS isoform could be contributing to the NOx present in the CSF or in the spinal cord interstice in both normal rats and those with CYP-induced cystitis? Although we did not use selective inhibitors for each of the isoforms, considering that the NOS II isoform is not expressed in the cells of the Central Nervous System under normal conditions, the most probable answer is that, in animals without cystitis, metabolites of the NO derive from NO produced by constitutive NOS (NOS I or NOS III). In relation to NO metabolites present in animals with cystitis, which were elevated in the spinal cord interstice and in CSF, these most probably derive also from NO produced by the type I NOS synthase, since we have shown previously that it is this isoform that is increased in the spinal neurons of rats with chemically induced cystitis^(5)^; in addition it has been consistently observed that during a peripheral inflammatory process, it is also this isoform that is most increased in the spinal cord segments receiving the afferents from the inflamed region^(16)^.

It is noteworthy that the concentration of NOx observed in microdialysate samples of the CSF in normal animals under baseline conditions was similar to that reported by Kawamata and Omote^(10)^; similarly, the baseline values of NOx in the microdialysate of the spinal cord interstice in normal animals were comparable to those reported by Wu et al.^(9)^ in the L5 segment and by Vetter et al.^(11)^ in the L4 segment.

Another observation of the present study, no less intriguing, is the fact that the non-selective NOS inhibitor, L-NMMA, although able to decrease significantly the concentration of NOx both in the CSF and in the spinal cord interstice in both groups of animals, only partially reduced NOx levels, even after a supramaximal concentration. It is interesting to note that similar results were reported by Kawamata and Omote^(10)^ in their experiments with probes implanted in the subarachnoid space using a greater concentration of L-NMMA (10mM), and by Yamada and Nabeshima^(17)^ in their experiments with a probe implanted in the rat cerebellum using 1.0mM of L-NMMA. Considering that supramaximal concentrations of L-NMMA were used, a simple interpretation of this finding could be that the concentration of NOx present in the CSF and in the spinal cord interstice (or in the cerebellum) not only reflects the real time production of NO synthesized de novo by NO synthasesS, but also the metabolites of NO synthesized previously, or NO derived from NO-containing compounds other than nitrite and nitrate, such as for example S-nitrosothiols^(18)^.

## CONCLUSION

In summary, this study shows that NO metabolites are present in the subarachnoid space and in the dorsal horns interstice of spinal cord L6-S1segments in normal rats as well as in rats with CYP-induced cystitis, and that the bladder inflammation is associated with an increase in the production of NO in these spinal cord segments. Results of this study also indicate that in both circumstances, the inhibitor L-NMMA is not able to completely reduce the production of the NO metabolites, suggesting that there is the possibility of an alternative pathway to that of the NOS enzyme which is responsible for the production of NO that generates these metabolites.

## References

[1] Fowler CJ, Griffiths D, de Groat WC (2008). Neural control of micturition. Nat Rev Neurosci.

[2] Rice AS (1995). Topical spinal administration of a nitric oxide synthase inhibitor prevents the hyperreflexia associated with a rat model of persistent visceral pain. Neurosci Lett.

[3] Kakizaki H, de Groat (1996). Role of spinal nitric oxide in the facilitation of the micturition reflex by bladder irritation. J Urol.

[4] Callsen-Cencic P, Mense S (1997). Expression of neuropeptides and nitric oxide synthase in neurons innervating the inflamed rat urinary bladder. J Auton Nerv Syst.

[5] Lagos P, Ballejo G (2004). Role of spinal nitric oxide synthase-dependent processes in the initiation hyperreflexia associated with cyclophosphamide-induced cystitis. Neuroscience.

[6] Sluka KA, Westlund KN (1992). An experimental arthritis in rats: dorsal horn aspartate and glutamate increases. Neurosci Lett.

[7] Rocha JN, Smith C, Chancellor MB, de Groat WC, Boone T (2003). Changes in spinal transmitters release patterns during volume or noxious evoked bladder afferent activity. J Urol.

[8] Souza-Filho MV, Lima MV, Pompeu mm, Ballejo G, Cunha FQ (1997). Involvement of Nitric Oxide in the pathogenesis of cyclophosphamide-induced hemorrhagic cystitis. Am J Pathol.

[9] Wu J, Lin Q, Lu Y, McAdoo D, Willis WD (1998). Nitric oxide contributes to central sensitization following intradermal injection of capsaicin. Neuroreport.

[10] Kawamata T, Omote K (1999). Activation of spinal N-methyl-D-Aspartate receptors stimulates a nitric oxide/cyclic guanosine 3'-5'-monophosphate/glutamate release cascade in nociceptive signaling. Anesthesiology.

[11] Vetter G, Geisslinger G, Tegeder I (2001). Release of glutamate, nitric oxide and prostaglandin E2 and metabolic activity in the spinal cord of rats following peripheral nociceptive stimulation. Pain.

[12] Watanabe C, Sakurada T, Okuda K, Sakurada C, Ando R, Sakurada S (2003). The role of spinal nitric oxide and glutamate in nociceptive behaviour evoked by high-dose intrathecal morphine in rats. Pain.

[13] Maggi CA, Lecci A, Santicioli P, Del Bianco E, Giuliani S (1992). Cyclophosphamide cystitis in rats: involvement of capsaicin-sensitive primary afferents. J Auton Nerv Syst.

[14] Applebaum AE, Vance WH, Coggeshall RE (1980). Segmental localization of sensory cells that innervate the bladder. J Comp Neurol.

[15] Ruscheweyh R, Goralczyk A, Wunderbaldinger G, Schober A, Sandkühler J (2006). Possible sources and sites of action of the nitric oxide involved in synaptic plasticity at spinal lamina I projection neurons. Neuroscience.

[16] Schmidtko A, Tegeder I, Geisslinger G (2009). No NO, no pain? The role of nitric oxide and cGMP in spinal pain processing. Trends Neurosci.

[17] Yamada K, Nabeshima T (1997). Two pathways of nitric oxide production through glutamate receptors in the rat cerebellum in vivo. Neurosci Res.

[18] Chvanov M, Gerasimenko OV, Petersen OH, Tepikin AV (2006). Calcium-dependent of NO from intracellular S-nitrosothiols. EMBO J.

